# Tropheryma whipplei infection in the lung of a patient with long COVID: a case report

**DOI:** 10.1186/s12879-024-09183-6

**Published:** 2024-03-06

**Authors:** Wenjing Ruan, Jing Xu, Fan Yang, Xiaohong Wu, Kejing Ying

**Affiliations:** 1 Regional Medical Center for the National Institute of Respiratory Disease, Sir Run Run Shaw Hospital, School of Medicine, Zhejiang University, 3 East Qingchun Road, 310016 Hangzhou, China; 2 Department of Pathology, Sir Run Run Shaw Hospital, School of Medicine, Zhejiang University, Hangzhou, China

**Keywords:** Tropheryma whipplei, Long COVID, Lung

## Abstract

**Background:**

Immune dysregulation in individuals with long COVID has been detected. Differential diagnosis of diffuse infiltration on chest CT in long COVID is challenging.

**Case presentation:**

A 62-year-old man presented with a 10-month history of dyspnea after COVID-19 infection. Dyspnea became worse in the one month preceding presentation. The chest CT showed multifocal, subpleural, bilateral opacities due to long-COVID, and infiltration around the bronchovascular bundle in the bilateral lower lung field. The pathology for the transbronchial cryobiopsy (TBCB) first reported chronic inflammation (mainly interstitial pneumonia). The patient had positive results on tests for the antibody, RO-52+, EJ+. The presumptive diagnosis of connective tissue disease-interstitial lung disease was made. Prednisone and cyclophosphamide were given. At follow-up one month later, the chest CT showed new diffuse ground-glass infiltration. The previous TBCB specimen was re-evaluated. Foamy macrophages were found in the alveolar air space. Periodic acid-Schiff (PAS) staining was performed. Numerous intracytoplasmic organisms were detected, with morphologic features consistent with those of *Tropheryma whipplei*. The patient recovered after intravenous ceftriaxone and oral trimethoprim-sulfamethoxazole. The final diagnosis was lung *T. whipplei* infection and long COVID-19.

**Conclusion:**

This is the first case report of *Tropheryma whipplei* infection in the lung of a patient with long COVID-19. *T. whipplei* should be considered as a potential pathogen for diffuse lung infiltration in the post-COVID-19 era.

## Background

It is undeniable that pulmonologists encounter diagnostic difficulty when interpreting diffuse infiltration on chest computed tomography (CT) scans. The most important step is to clarify the etiology of the diffuse infiltration of the lung– whether it is infection, interstitial lung disease, cancer, or another less common disorder. For occult infections, pathogens such as Mycobacterium, *Pneumocystis carinii*, fungi and Nocardia are generally considered.

The situation is more complicated in the post-COVID-19 era. Some individuals can not recover from COVID-19. Long COVID is a multi-systemic condition often comprising severe symptoms that follow COVID-19 infection [1]. Some patients suffer from lung fibrosis as a complication [2, 3]. Air trapping has been demonstrated in several chest CT imaging studies in long COVID-19 patients [4]. Furthermore, occult pulmonary infection may increase in long COVID-19 patients owing to the weakened immunity of the airway and alveolar epithelial cells.

Whipple’s disease is a rare, chronic, multi-systemic infection caused by *Tropheryma whipplei*. Weight loss, arthralgia and diarrhea are the common symptoms [5, 6]. Whipple’s disease presenting with lung parenchymal involvement with or without gastrointestinal (GI) symptoms has been reported [7–12]. Common symptoms included dyspnea and dry cough, and findings on chest imaging which include pulmonary nodules, pulmonary cavities, interstitial patterns, pleural effusions and mediastinal lymphadenopathy. Colonization by *T. whipplei* has also been reported [13]. Here, we report a case of *T. whipplei* infection of the lung in a patient with long COVID.

## Case presentation

A 62-year-old man presented with a 10-month history of dyspnea after COVID-19 infection. Dyspnea became worse in the month preceding presentation. Ten months earlier, he had taken a trip to Australia to visit his daughter, where he became infected with COVID-19. He had a fever and a slight cough. He had difficulty in breathing when he took a 30-minute walk. He took over-the-counter medication for a common cold; this did not include paxlovid or cortisone. The symptoms of fever and cough lessened. However, there was no reduction in the dyspnea; he sought no medical care for this symptom. For the one month preceding presentation, he found difficulty in breathing after only a 10-minute walk. There was no fever, cough or sputum. The patient’s medical history included twenty years of hypertension. He took valsartan and hydrochlorothiazide capsules 125 mg per day and amlodipine besylate 5 mg per day. The patient had given up smoking and drinking for one year. He had no pets. Two years ago, he retired, and had had no contact with any toxic environment.

His vital signs were within normal limits. Oxygen saturation was 89% at room air. Auscultation revealed crackles in the lower lung fields. No lymph nodes were palpable. Heart and abdomen were unremarkable. The patient denied intermittent fever, rash, dry eye, dry mouth, Raynaud’s phenomenon, arthritis or symmetrical weakness of the extremities. No skin lesions (heliotrope rash, Gottron’s papules, shawl sign, mechanical hands) were found.

Chest imaging, spirometry and blood examination were ordered to assess parenchymal lung disease. White-cell count was 10 × 10^3^/µL (neutrophils 7.15 × 10^3^/µL, lymphocytes 1.76 × 10^3^/µL, and eosinophils 0.35 × 10^3^/µL). Hemoglobin level was 12.9 g per deciliter; platelet count was 400 × 10^3^ /µL; C-reactive protein level was raised (31.8 mg/L). Liver enzymes and creatinine levels were normal. The patient had a restrictive defect on spirometry. The forced expiratory volume in one second (FEV1) was 61.4% of predicted. The forced vital capacity (FVC) was 51.2% of predicted. The FEV1/FVC ratio was 81.56%, i.e., in the normal range. The total lung capacity (TLC) was 62.3% of predicted. The diffusing capacity of the lung for carbon monoxide (DLCO) was low, 42.1% of predicted.

**Fig. 1:**
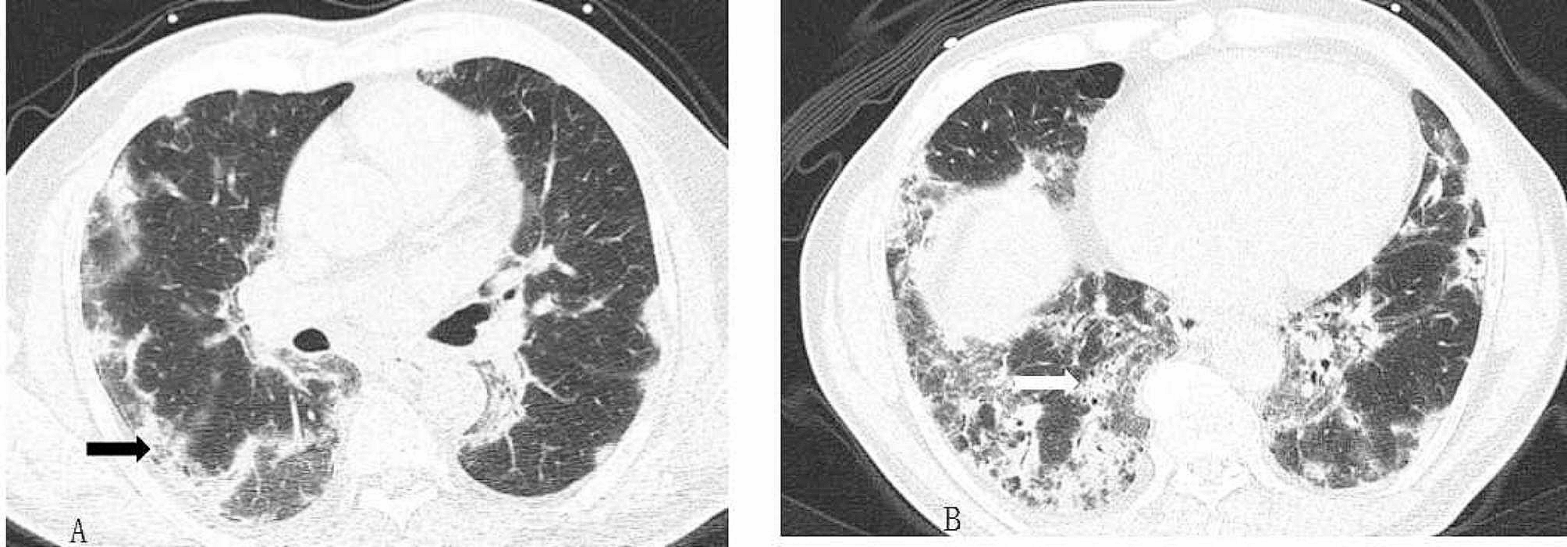
**A**-**B**, chest CT scan revealing multiple multifocal, subpleural, bilateral opacities (black arrow, **A**) and infiltration around the bronchovascular bundle in the bilateral lower lung field (white arrow, **B**)

The chest CT of the patient presented with a complex constellation of features (Fig. 1). The infiltration as seen on CT consisted of two components: multifocal, subpleural, bilateral opacities; and the infiltration around the bronchovascular bundle in the bilateral lower lung field. Some individuals who had been infected with COVID-19 could experience long-term effects from their infection, known as long COVID. Empirically, the subpleural bilateral opacities were interpreted as being due to long COVID [14].

With regard to the cause of the infiltration around the bronchovascular bundle, the following observations were made. The sample from the patient’s mouth tested negative for COVID-19 using PCR. He tested positive for the antibody, RO-52+, EJ+. Transbronchial cryobiopsy (TBCB) was performed on the right lower lobe posterior segmental bronchus. The biopsy reported chronic inflammation (mainly interstitial pneumonia). *T. whipplei* was the sole possible pathogen detected in the bronchoalveolar lavage fluid(BALF)(670 reads) by next-generation sequencing technology (NGS). BALF contained 800 cells/µL (64% lymphocytes, 22% neutrophils, 10% macrophages, 4% eosinophils).

The concentrations of skeletal muscle enzymes were normal. He was assessed by a rheumatologist and a presumptive diagnosis of connective tissue disease-interstitial lung disease (CTD-ILD) was made.

The significance of *T. whipplei* detection in BALF still needed to be considered, in terms of whether this involved a carrier or a pathogen. There was no fever, cough or sputum. The pulmonologists initially concluded that *T. whipplei* was colonized in BALF. Prednisone and cyclophosphamide were given.

**Fig. 2:**
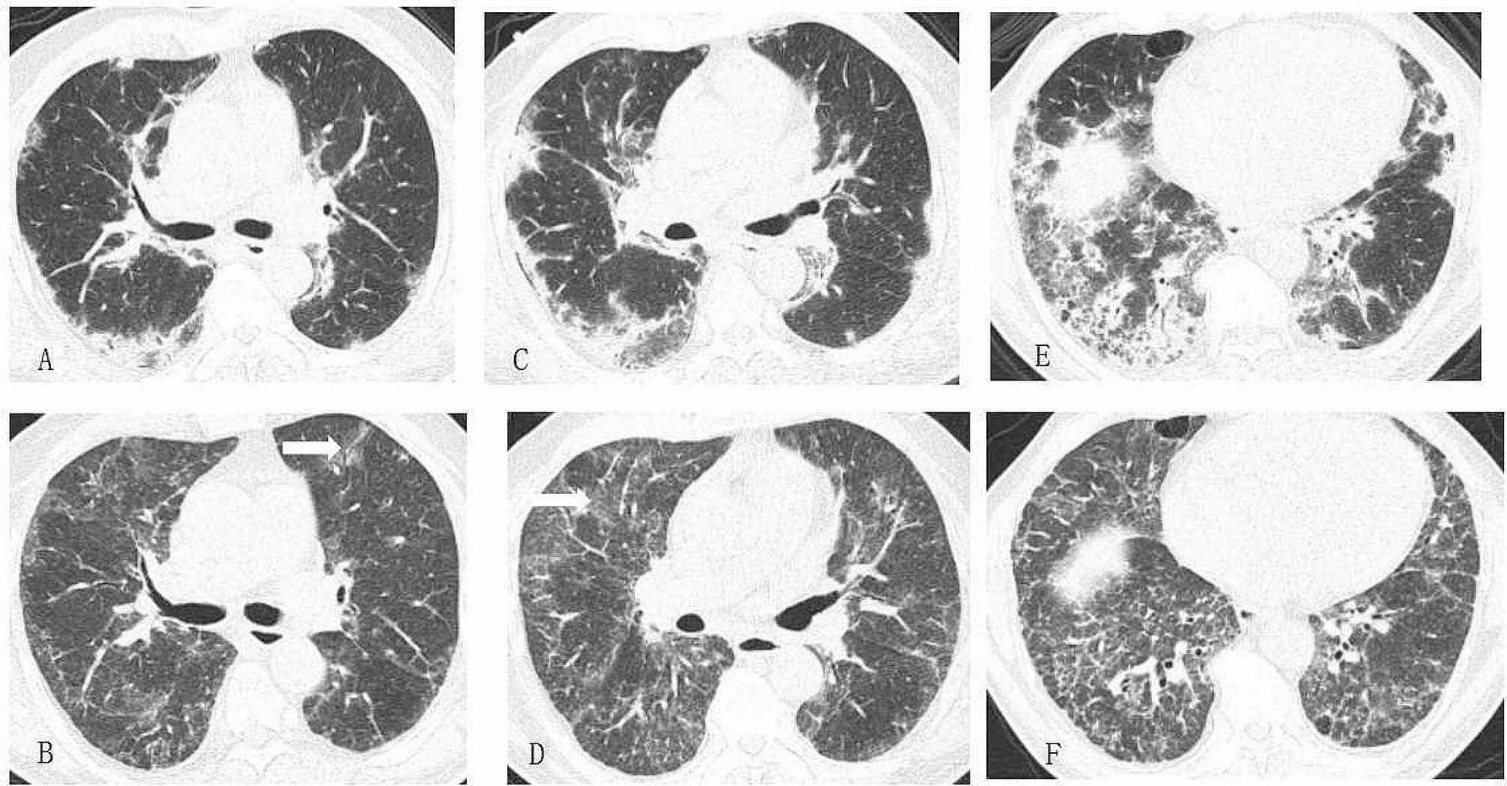
Chest CT at initial diagnosis is shown in **A**, **C** and **E**. Chest CT scan after prednisone treatment for one month (**B**, **D**, **F**) revealed partial absorption of the opacities. New diffuse ground-glass infiltration along bronchovascular bundles (white arrow)in bilateral lung field

At follow-up one month later, the patient complained that the dyspnea had only improved a little. He had a low fever 3 days earlier. Oxygen saturation was still low, at 89%. The C-reactive protein level was 28.0 mg/L. A CT scan of the chest showed partial absorption of the opacities. Most of the subpleural opacities due to long COVID resolved after prednisone treatment. New diffuse ground-glass infiltration along bronchovascular bundles in bilateral lung field occurred (Fig. 2). The recent fever and the new diffuse infiltration on CT suggested an infection.

**Fig. 3:**
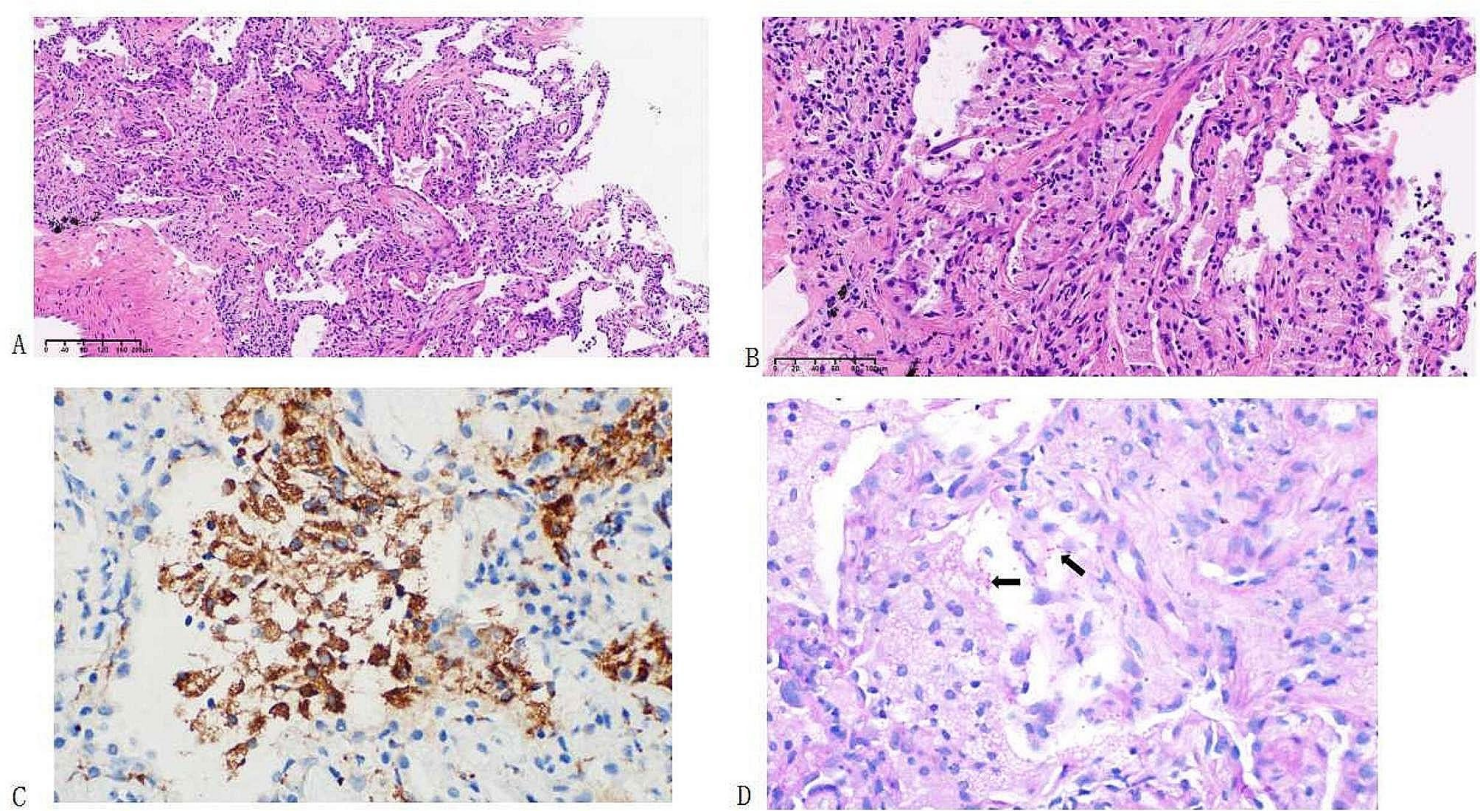
Hematoxylin and eosin staining (**A**: low-power photomicrograph; **B**: original magnification×100) showing the interstitial pneumonia with lymphocyte and plasmacyte infiltration of the alveolar septum and focal proliferation of fibrous tissue. Numerous foamy macrophages are seen in the alveolar air space. The foamy macrophages stained positive for CD68 by immunohistochemistry (**C**). Periodic acid-Schiff staining showing numerous intracytoplasmic organisms (black arrows), with morphologic features consistent with *T. whipplei* (original magnification× 200 (**D**)

The clinical suspicion for the earlier diagnosis of CTD-ILD therefore had to be re-considered. The pathogen in the BALF, *T. whipplei*, was then considered as a potential contributing factor. On pathological re-evaluation of the TBCB specimen, foamy macrophages in the alveolar air space were found, and periodic acid-Schiff (PAS) staining revealed numerous intracytoplasmic organisms, with the morphologic features consistent with *T.whipplei* (Fig. 3). Lung *T. whipplei* infection was therefore considered responsible for the symptoms in this case. Cyclophosphamide was discontinued and the dosage of prednisone rapidly reduced. The patient received intravenous ceftriaxone for two weeks. Oral trimethoprim–sulfamethoxazole at a dose of 160 mg of trimethoprim and 800 mg of sulfamethoxazole twice daily was prescribed.

**Fig. 4:**
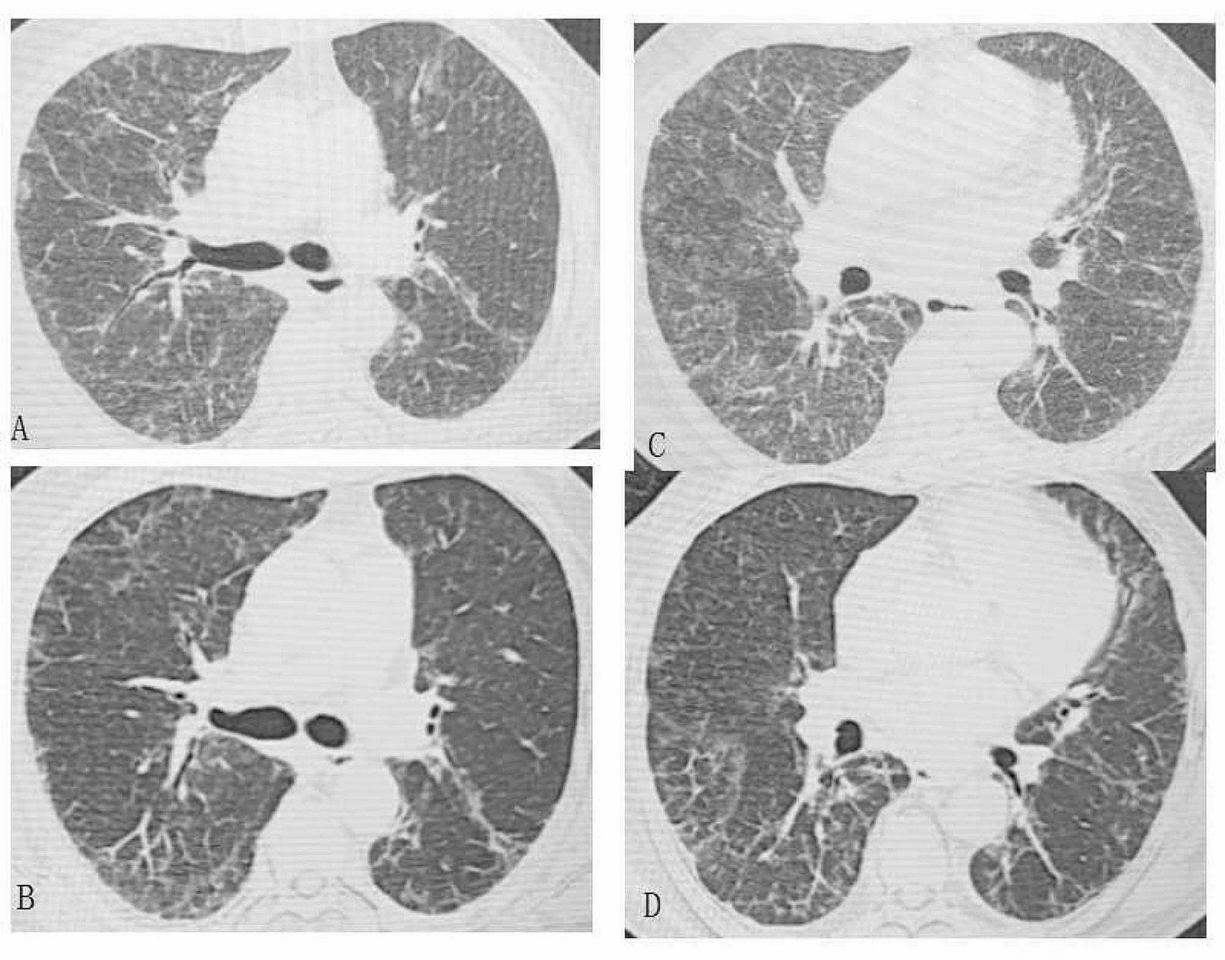
The CT scan after antibiotic administration (**B**, **D**) shows the diffuse infiltration was partially absorbed. The lung field was clearer than before. Chest CT scan before the antibiotics is showed in **A** and **C**

One month later, with a gradually reducing dose of prednisone to 5 mg per day, the patient returned to the pulmonology clinic and reported an obvious resolution of dyspnea. Oxygen saturation was raised to 93%, and the CT scan showed only a few fibrous cords and reticular opacities (Fig. 4). C-reactive protein level was normal, at 4.5 mg/L, and the erythrocyte sedimentation rate was lower than before (24 mm/hr). The improvement of the restrictive lung function defect was demonstrated. The FEV1 was 67.7% of predicted. The FVC was 66.5% of predicted. The TLC was 66.5% of predicted. The DLCO was 50.7% of predicted. The trimethoprim-sulfamethoxazole treatment was continued; the prednisone discontinued.

Two months later, in the pulmonology clinic, the patient reported complete relief of his earlier breathing difficulties; he could routinely take a walk of several kilometers per day.

## Discussion and conclusions

Our case is unusual and educational. This is the first case report of lung *T. whipplei* infection in a patient with long COVID-19. Shortness of breath and cough are the most common respiratory symptoms in long COVID-19 patients [1]. In the present case, the sole symptom for long COVID-19 was dyspnea. The multifocal, subpleural, bilateral opacities on the chest CT could explain the cause of dyspnea. After 9 months, dyspnea became worse and the opacities on the chest CT image progressed. The management increased our understanding of lung *T. whipplei* infections in long COVID patients. *T whipplei* should be considered as a potential pathogen for diffuse lung infiltration.

Owing to the overlap of features with those of many chronic inflammatory diseases and the difficulty in detecting the pathogen, cases of *T. whipplei* infection have been treated empirically with glucocorticoids [6, 11]. In the present case, the detection of *T. whipplei* in the TBCB specimen was delayed. The H&E stain of the specimen revealed two principal features: one was the thickened alveolar wall infiltrated with inflammatory cells and fibrous tissue; the other was numerous foamy macrophages in the alveolar space. However, a pathological diagnosis of chronic inflammation (mainly interstitial pneumonia) was provided initially. No special staining was performed to detect the possible presence of microorganisms. As both infectious and non-infectious factors could lead to the aggregation of foamy macrophage cells in the alveolar air space ( [15, 16]), special staining should be performed to detect the possible presence of microorganisms.

Lin et al. analyzed 1,725 BALF samples by NGS. Seventy BALs (70/1725, 4.0%) from 70 patients were positive for *T. whipplei*. Only nine patients (12.9%) were immunocompromised [17]. For non-immunocompromised patients, the detection of *T. whipplei* needs to be considered. The spectrum of the pathogenic effects of *T. whipplei* in BALF is broad. *T. whipplei* can be an etiologic pathogen in acute respiratory infections. Some individuals are unable to clear the bacterium leading to persistence and asymptomatic carriage [5]. In the studies conducted by Guo et al., BALF of three ILD patients of unknown cause were evaluated via Nanopore sequencing [18]. *T. whipplei* was the sole or most abundant pathogen in all patients, comprising 1,385, 826, and 285 reads. The case highlights that it is essential to consider the possibility of *T. whipplei* infection as a diagnosis in ILD of unknown cause.

Auto-antibodies are cornerstone biomarkers in autoimmune diseases. However, the potential clinical significance of antibodies should be considered [19, 20]. Murng et al. reported that twenty-three patients (24%) with anti Ro52 + Ro60 − antibodies have no autoimmune features; the positive predictive value (PPV) for autoimmune disease was 73% (71/97) and 87% (87/100) for Ro52 + Ro60 − and Ro52 + Ro60+, respectively [19]. In the studies conducted by Wu et al., of the 4,782 cases testing positive for anti-Ro52 antibodies in the serum, 2/3 were diagnosed as CTD [20]. In our case, without typical symptoms and signs of CTD, only the positive detection of RO-52 and EJ-1 antibodies could not get to the diagnosis of CTD. The presence of RO-52 and EJ-1 warrant long-term follow-up.

The harmful side-effects of antibiotics have attracted attention, while we should also be aware of the hazards of cortisone over-use, especially in the COVID-19 era. As a result of over-using cortisone, we are gradually seeing a rise in infections, with obvious health dangers. For this patient, after reconsideration, we discontinued cyclophosphamide, and hastened the speed of corticosteroid withdrawal.

The COVID-19 virus damaged the bronchial mucosal barrier and the alveolar spaces, which increased the chance of infection. The increased number of cases of opportunistic infections in COVID-19 patients raises an important concern. The study conducted by Gangneux et al. showed the high prevalence of invasive pulmonary aspergillosis and candidemia in mechanically ventilated patients with COVID-19 [21]. Among long COVID-19 patients, especially for those with underlying diseases and who received immunosuppressive therapy, fungal infections account for most of the case reports. Viral, bacterial, protozoal and helminth infections were the other reported pathogens [22–25]. Our patient suffered from lung *T. whipplei* infection 9 months after the initial COVID-19 infection, and did not receive earlier immunosuppressive therapy. The impairment of the immune system function in long COVID-19 may be the main cause for the *T. whipplei* infection. Studies on immune dysregulation in individuals with long COVID have detected exhausted T cells, reduced CD4 + and CD8 + effector memory cell numbers and elevated PD1 expression on central memory cells, persisting for at least 13 months [1, 26, 27]. The pathogenesis of lung T. whipplei infection in patients with long COVID-19 should be investigated further.

## Data Availability

Not applicable.

## References

[1] Davis HE, McCorkell L, Vogel JM, Topol EJ (2023). Long COVID: major findings, mechanisms and recommendations. Nat Rev Microbiol.

[2] Hirawat R, Jain N, Aslam Saifi M, Rachamalla M, Godugu C (2023). Lung fibrosis: Post-COVID-19 complications and evidences. Int Immunopharmacol.

[3] Wiersinga WJ, Rhodes A, Cheng AC, Peacock SJ, Prescott HC (2020). Pathophysiology, transmission, diagnosis, and treatment of Coronavirus Disease 2019 (COVID-19): a review. JAMA.

[4] Cho JL, Villacreses R, Nagpal P, Guo J, Pezzulo AA, Thurman AL, Hamzeh NY, Blount RJ, Fortis S, Hoffman EA (2022). Quantitative Chest CT Assessment of Small Airways Disease in Post-acute SARS-CoV-2 infection. Radiology.

[5] Boumaza A, Ben Azzouz E, Arrindell J, Lepidi H, Mezouar S, Desnues B (2022). Whipple’s disease and Tropheryma whipplei infections: from bench to bedside. Lancet Infect Dis.

[6] Durand DV, Lecomte C, Cathebras P, Rousset H, Godeau P (1997). Whipple disease. Clinical review of 52 cases. The SNFMI Research Group on Whipple Disease. Societe Nationale Francaise De Medecine Interne. Med (Baltim).

[7] Kelly CA, Egan M, Rawlinson J (1996). Whipple’s disease presenting with lung involvement. Thorax.

[8] Winberg CD, Rose ME, Rappaport H (1978). Whipple’s disease of the lung. Am J Med.

[9] Hofmann P, Durisch N, Buetikofer C, Helmchen BM. Granulomatous lung disease and immune reconstitution inflammatory syndrome in Whipple’s disease. BMJ Case Rep 2021, 14(6). 10.1136/bcr-2021-243633 PMC8215230 34144955

[10] Urbanski G, Rivereau P, Artru L, Fenollar F, Raoult D, Puechal X (2012). Whipple disease revealed by lung involvement: a case report and literature review. Chest.

[11] Damaraju D, Steiner T, Wade J, Gin K, FitzGerald JM (2015). CLINICAL PROBLEM-SOLVING. A Surprising cause of chronic cough. N Engl J Med.

[12] Zhang WM, Xu L (2021). Pulmonary parenchymal involvement caused by Tropheryma whipplei. Open Med (Wars).

[13] Lozupone C, Cota-Gomez A, Palmer BE, Linderman DJ, Charlson ES, Sodergren E, Mitreva M, Abubucker S, Martin J, Yao G (2013). Widespread colonization of the lung by Tropheryma whipplei in HIV infection. Am J Respir Crit Care Med.

[14] Zheng Q, Lu Y, Lure F, Jaeger S, Lu P (2020). Clinical and radiological features of novel coronavirus pneumonia. J Xray Sci Technol.

[15] Shim D, Kim H, Shin SJ (2020). Mycobacterium tuberculosis infection-driven Foamy macrophages and their implications in tuberculosis control as targets for host-Directed Therapy. Front Immunol.

[16] Larsen BT, Chae JM, Dixit AS, Hartman TE, Peikert T, Roden AC (2019). Clinical and histopathologic features of Immune Checkpoint inhibitor-related Pneumonitis. Am J Surg Pathol.

[17] Lin M, Wang K, Qiu L, Liang Y, Tu C, Chen M, Wang Z, Wu J, Huang Y, Tan C (2022). Tropheryma whipplei detection by metagenomic next-generation sequencing in bronchoalveolar lavage fluid: a cross-sectional study. Front Cell Infect Microbiol.

[18] Guo Y, Li L, Li Z, Sun L, Wang H (2021). Tropheryma whipplei detection by Nanopore Sequencing in patients with interstitial lung disease. Front Microbiol.

[19] Murng SHK, Thomas M (2018). Clinical associations of the positive anti Ro52 without Ro60 autoantibodies: undifferentiated connective tissue diseases. J Clin Pathol.

[20] Wu S, Tang X, Wu L, Lu L, Feng X (2021). Anti-Ro52 antibodies in clinical practice: a single-centre experience. Int J Clin Pract.

[21] Gangneux JP, Dannaoui E, Fekkar A, Luyt CE, Botterel F, De Prost N, Tadie JM, Reizine F, Houze S, Timsit JF (2022). Fungal infections in mechanically ventilated patients with COVID-19 during the first wave: the French multicentre MYCOVID study. Lancet Respir Med.

[22] Rizvi SWA, Khan S, Shahbaz M, Gounder MS, Saif M, Khalid S (2023). Long-term outcomes of transcutaneous retrobulbar amphotericin B in COVID-19-associated mucormycosis. Indian J Ophthalmol.

[23] Chong WH, Saha BK, Neu KP (2022). Comparing the clinical characteristics and outcomes of COVID-19-associate pulmonary aspergillosis (CAPA): a systematic review and meta-analysis. Infection.

[24] Krivosikova L, Kuracinova T, Martanovic P, Hyblova M, Kaluzay J, Uhrinova A, Janega P, Babal P. Long COVID complicated by Fatal Cytomegalovirus and aspergillus infection of the lungs: an autopsy case report. Viruses 2023, 15(9). 10.3390/v15091810 PMC10535245 37766216

[25] Abdoli A, Falahi S, Kenarkoohi A (2022). COVID-19-associated opportunistic infections: a snapshot on the current reports. Clin Exp Med.

[26] Glynne P, Tahmasebi N, Gant V, Gupta R (2022). Long COVID following mild SARS-CoV-2 infection: characteristic T cell alterations and response to antihistamines. J Investig Med.

[27] Klein J, Wood J, Jaycox JR, Dhodapkar RM, Lu P, Gehlhausen JR, Tabachnikova A, Greene K, Tabacof L, Malik AA (2023). Distinguishing features of long COVID identified through immune profiling. Nature.

